# ReHypar: A Recursive Hybrid Chunk Partitioning Method Using NAND-Flash Memory SSD

**DOI:** 10.1155/2014/658161

**Published:** 2014-04-03

**Authors:** Jaechun No, Sung-Soon Park, Cheol-Su Lim

**Affiliations:** ^1^College of Electronics and Information Engineering, Sejong University, 98 Gunja-Dong, Gwangjin-Gu, Seoul 143-747, Republic of Korea; ^2^Department of Computer Engineering, Anyang University and Gluesys Co. LTD, Anyang 5-Dong, Manan-Gu, Anyang 430-714, Republic of Korea; ^3^Department of Computer Engineering, Seokyeong University, 16-1 Jungneung-Dong, Sungbuk-Gu, Seoul 136-704, Republic of Korea

## Abstract

Due to the rapid development of flash memory, SSD is considered to be the replacement of HDD in the storage market. Although SSD retains several promising characteristics, such as high random I/O performance and nonvolatility, its high expense per capacity is the main obstacle in replacing HDD in all storage solutions. An alternative is to provide a hybrid structure where a small portion of SSD address space is combined with the much larger HDD address space. In such a structure, maximizing the space utilization of SSD in a cost-effective way is extremely important to generate high I/O performance. We developed ReHypar (recursive hybrid chunk partitioning) that enables improving the space utilization of SSD in the hybrid structure. The first objective of ReHypar is to mitigate the fragmentation overhead of SSD address space, by reusing the remaining free space of I/O units as much as possible. Furthermore, ReHypar allows defining several, logical data sections in SSD address space, with each of those sections being configured with the different I/O unit. We integrated ReHypar with ext2 and ext4 and evaluated it using two public benchmarks including IOzone and Postmark.

## 1. Introduction

Over decades, the file system researches have been concentrated on reducing the mechanical positioning overhead of hard disks, such as seek time. However, as the technology of flash memory is growing these days, the storage market is being attracted to SSD (solid-state device) due to its promising characters, such as nonvolatility, reliability, and absence of seek time. However, besides the high expense ratio per storage capacity as compared to HDD [1, 2], SSD suffers from the serious weaknesses in replacing HDD to build the large-scale storage solutions.

The drawbacks of SSD include the erase overhead before write operations [3, 4] and wear-leveling to evenly distribute I/O requests among flash blocks [5–7]. Several researches have been performed to alleviate such SSD weaknesses either by implementing flash memory-specific file systems [8–13] or by utilizing the inside SSD structure such as FTL [5, 14, 15]. Among them, most flash memory-specific file systems or FTL have adopted the sequential log structure that was originated from the log-structured file system [16], to reduce the erase overhead of flash memory.

Although the file update behavior of the log-structured file system is appropriate for flash memories, its sequential log structure can cause the significant performance overhead in locating valid blocks [11]. Also, the optimization using SSD data structures or modules can contribute to reducing its semiconductor overhead, but such a method cannot easily be used in the commercial SSDs because most SSD providers rarely reveal SSD internals.

In this paper, we present a new form of hybrid data allocation scheme, called ReHypar (recursive hybrid chunk partitioning), which can be used in the hybrid structure whose address space is organized by integrating a small portion of NAND-flash SSD partition with the much larger HDD partition. In ReHypar, file accesses in SSD partition are executed on extent-basis and also file updates are performed in-place, which differs from log-structured file systems. Furthermore, ReHypar does not require using SSD internal data structures or modules, except for flash block size. The contribution of ReHypar is as follows.

- ReHypar uses the flexible data layout in such a way that SSD address space is partitioned into multiple, logical data sections, with each of those data sections being composed of the different extent size. Such a space configuration enables mapping files to the appropriate data sections, according to file size and usage, without affecting the directory hierarchy.
- ReHypar uses the chunk partitioning to allocate files in a fine-grained way. In the partitioning, extents are divided into a set of chunks and the remaining free spaces in the chunks after file allocations are further partitioned into the lower level. As a result, such free spaces can be reused for file allocations, while alleviating extent fragmentation overhead.
- Unlike the data collection using logs on FTL layer [5] or LRU-like data replacement on top of FTL [17, 18], the data coalescing scheme of ReHypar does not require having accesses to SSD internals. Given that the flash block size is known to users, ReHypar can align the extent size with flash block boundaries on top of VFS layer, in order to reduce the write and erase costs in flash memory.

This paper is organized as follows. In Section 2, we discuss the related studies. In Section 3, we describe the overall structure and I/O optimizations of ReHypar. In Section 4, we present the performance measurements of ReHypar while comparing them to those of ext2, ext4, and xfs. Finally, in Section 5, we conclude with a summary.

## 2. Related Studies

Recently, many studies have been performed to overcome SSD disadvantages. Although SSD has several promising potentials including high random I/O performance, its erase-before-write behavior is the main obstacle in producing good I/O bandwidth, because the time for erasing a flash block is several orders of magnitude higher than that for I/O. Also, repeatedly erasing flash blocks causes the worn-out blocks, resulting in the data loss. Therefore, evenly distributing the write requests among flash blocks is inevitable to prolong the lifetime of SSD (100 K for SLC, 10 K for MLC [19]).

Several researches have been performed to reduce the erase-before-write overhead, either by using LRU-like replacement in SSD internal memory or by introducing the new form of FTL. In SSD, I/O operations are performed per page and the mapping between logical address and physical address can be performed in blocks or pages. Although the page mapping [20, 21] is faster than the block mapping, it requires having the larger mapping table. On the contrary, the time for mapping with blocks is slower because of page-copy operations. In the mapping using log blocks [18], several log blocks are prepared to merge write operations to the same location, to avoid repeatedly writing data to the same position. However, due to its small number of log blocks to be reserved, it can cause the frequent update of the flash block and low space utilization.

The fully associative section translation [22] tried to overcome the disadvantages of log block-based mapping, allowing log blocks to be used by pages belonging to any data blocks. However, it still suffers from the significant erase overhead in random write operations. Also, several LRU-like algorithms have been implemented to reduce the write cost to flash memory, by delaying write operations to flash blocks. For example, CFLRU [23] maintained the LRU-like page linked list in memory. Before writing to flash memory, the modified page is inserted into the linked list until it is selected as a victim. Also, the selection for a victim is first performed in the clean-first region to reduce the erase cost. LRU-WSR [24] is much like the second-chance replacement algorithm in which the flush-out to flash memory is performed by checking the cold-flag. Delaying write operations to flash memory is postponed by clearing the cold-flag, while moving dirty pages to MRU position to give them the second chance before writing to flash memory.

On the other hand, FAB [25] and BPLRU [17] are all worked per block for the eviction from memory. For example, FAB selects a block containing the large number of pages as a victim, expecting to switch the entire pages of a data block to the new ones in flash memory. BPLRU is also worked in block while choosing a block maintaining the large number of pages in LRU list. However, it is optimized for random write operations by using the write buffer inside SSD.

Most methods mentioned require using SSD internals, such as FTL or replacement buffer containing page or block information. However, such information is rarely available to users in the commercial SSDs. Our method does not need to access SSD data structure or modules, except for flash block size if available.

Several researches have been performed to optimize the erase cost by introducing the log-structured concept in the file system level. The out-of-place I/O behavior in the log-structured file system differs from the in-place I/O behavior in legacy file systems such as ext2/4. Since such an out-of-place I/O behavior is appropriate for flash memory, most flash file systems introduced the log-structured method in their concept. For example, JFFS2 [12] used logs to be organized in variable-length nodes, to merge dirty data. Each node maintains file metadata including file name, inode number, and a range of data. Also, YAFFS [9] used logs in the fixed-size chunks. The head chunk with chunk number zero includes file metadata. However, both file systems need to scan the entire logs to organize the directory hierarchy at file mount time.

Conquest [26] and FlexFS [27] are all hybrid file systems where the address space of Conquest is constructed by integrating RAM with hard disk. However, the RAM used in Conquest does not require being erased before writing; therefore there is no need to consider the data alignment with blocks. On the contrary, the address space of FlexFS is organized by using only two kinds of SSDs: SLC and MLC. DFS [10] uses fusion-ioDrive [28] to provide the virtualized flash storage layer where the traditional block device is combined with FTL. The layer enables providing the direct data path between file system and the controller, while providing the thin software layer. However, such a layer can cause higher expenses than HDD and also porting the layer to other file systems may be difficult since it is tightly coupled with DFS.

ReHypar differs from flash-specific file systems in such a way that I/Os in ReHypar occur in-place. Also, it enables dividing SSD address space into several data sections, with each being configured with the different extent size.

## 3. Implementation Details

This section presents the overall structure and I/O optimizations of ReHypar.

### 3.1. Chunk Partitioning

ReHypar is the hybrid block allocation mechanism whose objective is to provide better storage utilization of the hybrid structure whose address space is constructed by integrating a small portion of SSD partition with the much larger HDD partition. In order to do that, ReHypar attempts to reduce the space fragmentation of SSD partition by reordering the remaining free space of I/O units (extents). The free space of an extent is recursively partitioned after file allocations, until either it is fully occupied with file data or the reordering time to be given to the extent is expired.

Furthermore, in ReHypar, SSD partition can be divided into several data sections. Each extent size of the data sections enables being configured with the different length so that files can be mapped into the appropriate data section according to their length, access pattern, and usage. Finally, given that the flash block size is known to ReHypar, the extent size enables being aligned with flash block boundaries on VFS layer, which might contribute to reducing the erase overhead on SSD partition without requiring the knowledge about SSD internals.

**Definition 1 (extent structure)**

ReHypar divides SSD partition into multiple data sections, with each of them being configured with the different extent size. Let *E* be the extent of a data section. Then, *E* is composed of (*s*, *v*, *L*, *C*, *ϕ*).

(1) *s* = 2^*n*^(*n* > 1) is the size of *E* in blocks.
(2) *v* is the threshold value in blocks.
(3) *L* is the partitioning level.
(4) *C* = {*c*_*L*_^*i*^ | (*H* = −1) ≤ *i* < log_2_ *s*, *L* ≥ 0} is a set of chunks at level *L*. The *i* denotes the chunk index. If *i* = *H*, then it implies the head chunk.
(5) *ϕ* is the partitioning function such that
  (1) $$\begin{array}{c} \phi \left(b,E\right)=\left\{c_{0}^{H},c_{0}^{0},c_{0}^{1},\ldots ,c_{0}^{(\text{lo}{\text{g}}_{2}s)-1}\right\},\;\text{if}\;L=0 \end{array}$$

*ϕ*(start(*c*_*L*_^*i*^), *c*_*L*_^*i*^) = {*c*_*L*+1_^*H*^,  *c*_*L*+1_^0^,…,  *c*_*L*+1_^*i*−1^}, if *L* > 0 and *i* ≥ log_2_ *v*.

In *ϕ*(*b*, *E*),  *b* denotes the block position where the partitioning begins.

**Figure 1:**
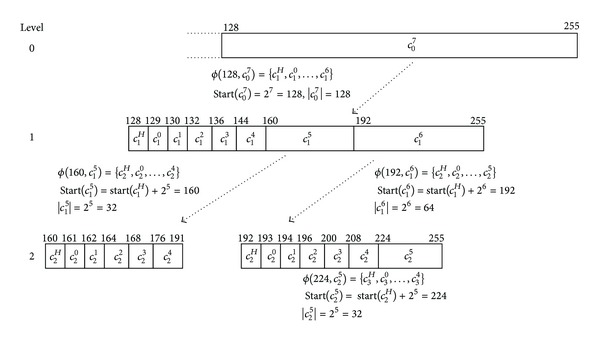
The chunk partitioning of ReHypar.

At the partitioning level zero, *E* is partitioned into (log_2_ *s*) + 1 chunks. If the size of chunks is larger than or equal to *v* (chunk index is log_2_ *v*), then chunks are recursively partitioned into the subsequent level until their size becomes smaller than *v*.  Figure 1 shows an example of the chunk partitioning. Also, let start(*c*_*L*_^*i*^) and |*c*_*L*_^*i*^| be the starting block position and the size in blocks of chunk *i* at level *L*. Then, start(*c*_*L*_^*i*^) and |*c*_*L*_^*i*^| are defined as follows:

(2) $$\begin{array}{c} L=0\text{:}\;\text{start}\left(c_{0}^{H}\right)=0,\;\left|c_{0}^{H}\right|=1\\ \forall c_{0}^{j}\in \phi \left(b,E\right),\;\text{start}\left(c_{0}^{j}\right)=2^{j},\;\left|c_{0}^{j}\right|=2^{j}\;\text{if}\;\left(j>H\right)\\ L>0\text{:}\;\;\forall c_{L}^{k}\in \phi \left(\text{start}\left(c_{L-1}^{i}\right),c_{L-1}^{i}\right),\\ \text{start}\left(c_{L}^{H}\right)=\text{start}\left(c_{L-1}^{i}\right),\;\left|c_{L}^{H}\right|=1\\ \text{start}\left(c_{L}^{k}\right)=\text{start}\left(c_{L}^{H}\right)+2^{k},\;\left|c_{L}^{k}\right|=2^{k}\;\;\text{if}\;\left(k>H\right). \end{array}$$

The starting block position and the length of the head chunk at level zero are 0 and 1, respectively. On the other hand, the starting block position of the head chunk *c*_*L*_^*H*^ at level *L* (> 0) is the same as that of the parent *c*_*L*−1_^*i*^, where the head chunk is partitioned into. The length of each chunk at a level is the multiple of two, except for the head chunk whose length is of one. As a result, the starting block position of each chunk becomes the starting block position of the head chunk plus its length in blocks.

Figure 1 shows an example of the chunk partitioning of ReHypar on an extent of size 256 in blocks. The threshold value *v* is set to 32; therefore if the length of a chunk is no less than 32, then the partitioning takes place in the subsequent level to reduce the extent fragmentation.

In Figure 1, chunk *c*_0_^7^ of the level zero is partitioned into 8 chunks in level one, starting from *c*_1_^*H*^ to *c*_1_^6^. The starting block position and the length are calculated as 128. Similarly, chunks *c*_1_^5^ and *c*_1_^6^ are further divided in the level two, due to their length larger than or equal to *v*. The starting block position of *c*_1_^5^ is the one of the head chunk *c*_1_^*H*^ plus its length 32. In level two, the chunk *c*_2_^5^ originated from *c*_1_^6^ is the only one whose length is at least *v*; therefore, the partitioning step is applied to *c*_2_^5^:

(3) $$\begin{array}{c} \phi \left(128,c_{0}^{7}\right)\overset{\text{level}\;1}{\to }\left\{c_{1}^{H},c_{1}^{0},c_{1}^{1},\ldots ,c_{1}^{6}\right\},\\ \phi \left(160,c_{1}^{5}\right)\overset{\text{level}\;2}{\to }\left\{c_{2}^{H},c_{2}^{0},c_{2}^{1},\ldots ,c_{2}^{4}\right\},\\ \phi \left(192,c_{1}^{6}\right)\overset{\text{level}\;2}{\to }\left\{c_{2}^{H},c_{2}^{0},c_{2}^{1},\ldots ,c_{2}^{5}\right\},\\ \phi \left(224,c_{2}^{5}\right)\overset{\text{level}\;3}{\to }\left\{c_{3}^{H},c_{3}^{0},c_{3}^{1},\ldots ,c_{3}^{4}\right\}. \end{array}$$

### 3.2. Chunk Mapping

In this section, we describe how the new file is mapped into the chunk to be stored in SSD partition. We designed the mapping scheme to reduce the fragmentation overhead, by reusing the remaining space of extents as much as possible. Also, by adopting the simple calculation to the mapping scheme, we tried to minimize the computation overhead in the chunk mapping.

**Definition 2 (chunk mapping)**

Let pos be the block position of *E*. Then, for all *c*_*L*_^*i*^  (*i* ≥ log_2_ *v*), function split is defined as follows:

(4) $$\begin{array}{c} \text{split}\left(c_{L}^{i},\text{pos}\right)=c_{L+1}^{k},\;\text{where}\;2^{k}\leq \text{pos}-\text{start}\left(c_{L}^{i}\right)<2^{k+1}. \end{array}$$

Let pos be the last block position of a file *f* allocated in *E*. If the remaining space of *E* is larger than or equal to *v*, then *E* is reused for further file allocations. Let *g* be the next file to be allocated in *E*. To calculate the starting block position of *g* on *E*, split is recursively executed at each level. Suppose that pos is mapped to *c*_*L*_^*i*^ at level *L* and the size of *c*_*L*_^*i*^ is not smaller than *v*. Then, split is executed on the chunk to go down to level *L* + 1. If pos is mapped *c*_*L*+1_^*k*^ at *L* + 1 partitioned from *c*_*L*_^*i*^ and the size of *c*_*L*+1_^*k*^ is less than *v*, then *g* is allocated from the next chunk *c*_*L*+1_^*k*+1^.

**Figure 2:**
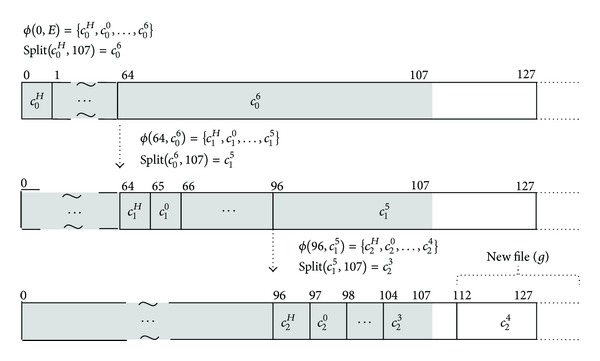
An example of chunk mapping.

Figure 2 shows an example of the chunk mapping on an extent *E*. Assume that a file was allocated from 0 to 107 block position. Applying split function on *E* produces *c*_0_^6^ that is the last chunk to be used for the allocation. Since the length of *c*_0_^6^ is larger than *v*(32), *c*_0_^6^ is partitioned to *c*_1_^*H*^, *c*_1_^0^,…, *c*_1_^5^ at level one and calculating split function with *c*_0_^6^ gives us *c*_1_^5^, which needs one more partitioning step to level two. The chunk *c*_2_^3^ where 107 is mapped is small than *v*; therefore no more chunk partitioning is needed. Also, the new file is allocated from *c*_2_^4^. Consider the following:

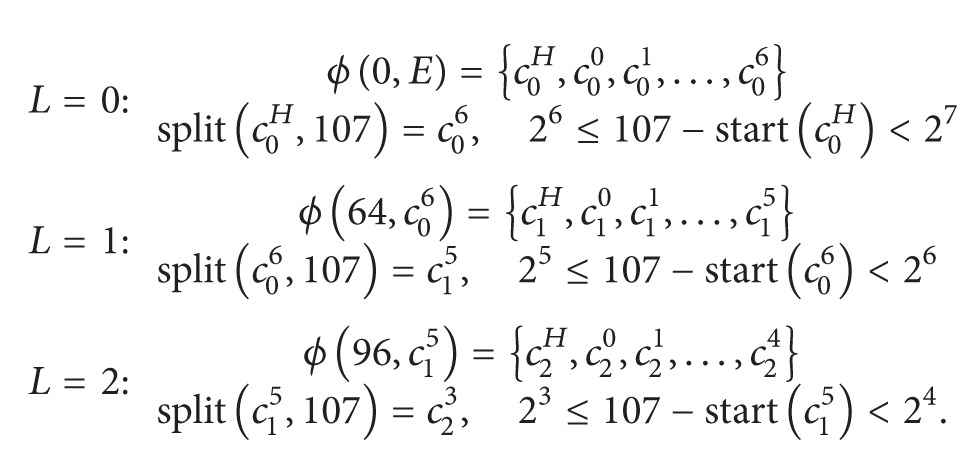
(5)


### 3.3. Allocation Algorithm

In ReHypar, each partitioning level *L* maintains a linked list of extents that contains a free space beginning from chunk *c*_*L*_^*i*^, in the decreasing order of the unused space size. At file system mount, ReHypar preserves a set of clean extents that are not yet used for file allocation. When clean extents are used for file allocation while leaving the unused space of at least *v*/2, the extent is connected to the appropriate linked list, according to the chunk index of free space. After either the entire chunks of extents are filled with data or the time for which extents are allowed to stay at memory for reuse is expired, the extent is written to SSD partition. If there are multiple extents available for reuse, then ReHypar chooses the extent that has been in memory the longest (*t*) and the distance (*d*) between the new file and files allocated to the extent is the closest.

Algorithm 1 shows the steps involved in the file allocation on extents. Let |*f*| be the size of a new file *f* and let *s* be the size of extents in blocks. In case that |*f*| ≥ *s*, choosing *m* clean extents in step 2 takes *O*(*m*). In step 4, the time complexity for calling MAP to reuse the last extent *E* is *O*(log_2_ *s*). Also, in step 6, connecting *E* to the appropriate linked list of *n* elements based on the chunk index of free space is *O*(*n*). Therefore, if the file size is at least the size of extent, then the time complexity for ALLOCATE is *O*(*m* + *n* + log_2_ *s*).

If the file size is less than the size of extent, then the file allocation is first performed to reuse extents in memory. In step 10, the algorithm checks the first extent of each linked list to see if there is an extent whose free space is large enough to allocate *f*. Since there are log_2_(*s*/*v*) levels and each level has (log_2_ *s*) + 1 chunks, the time for choosing an extent is *O*((log_2_ *s*)^2^). Since, in step 14, connecting *E* to the linked list of *n* elements takes *O*(*n*), the time complexity for ALLOCATE is *O*(*n* + (log_2_ *s*)^2^).

## 4. Performance Evaluation

We evaluated ReHypar on a server equipped with a 3 GHz quad-core Intel Xeon, 16 GB of main memory, two 720 GB of Seagate disk, and 80 GB of fusion-ioSSD ioDrive [28]. The operating system was CentOS release 6.2 with a 2.6.32 kernel. In ReHypar, we divided SSD partition into two data sections composed of 64 KB and 256 KB of extent sizes and mapped files to those data sections. To observe the effect of the chunk partitioning of ReHypar, we changed the threshold value (*v*) between 8 and 32.

In ReHypar, the extents with 64 KB of size are partitioned into seven chunks from *c*_0_^*H*^ to *c*_0_^5^ at level zero. On the other hand, the extents with 256 KB of size are partitioned into nine chunks from *c*_0_^*H*^ to *c*_0_^7^. With *v* = 8 in ReHypar (64 : 8) and ReHypar (256 : 8), the chunk partitioning to the lower level takes place in the case that a file is mapped from *c*_0_^3^. With *v* = 32, the partitioning occurs from *c*_0_^5^.  Table 1 shows the chunk partitioning on both extent sizes. The block size is 1 KB.

### 4.1. IOzone Experiments

**Figure 3:**
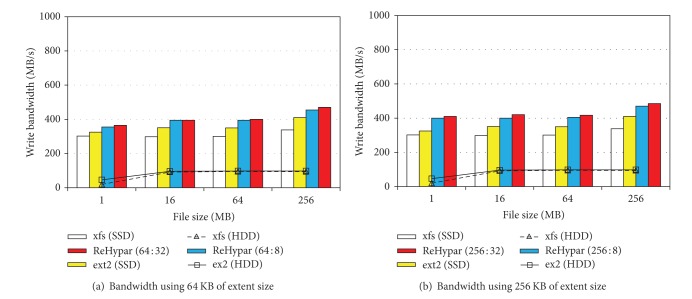
ReHypar write bandwidth as compared to ext2 and xfs.

We used IOzone benchmark with 8 KB of record size and −*e* option to invoke* fsync*(). Figures 3(a) and 3(b) show the write bandwidth of xfs, ext2, and ReHypar integrated with ext2. In Figure 3(a), the extent size is set to 64 KB and in Figure 3(b) the extent size is set to 256 KB. The figures show that ReHypar produces the higher I/O bandwidth over ext2 and xfs installed on HDD due to its hybrid structure.

In the figures, there are two aspects to be pointed out. First, due to the fact that ReHypar uses the large I/O granularity, it produces better I/O bandwidth as compared to ext2 and xfs installed on SSD. For example, with 256 MB of file sizes, ReHypar composed of 64 KB and 256 KB extent sizes gives 10% and 14% of performance speedup, respectively, over ext2 installed on SSD.

Second, changing the threshold value *v* from 8 to 32 does not affect I/O performance of ReHypar. For example, with 1 MB of file size, setting *v* from 8 to 32 does not show the noticeable change in both extent sizes. However, with 256 KB of extent size on top of 256 MB of file size, changing *v* from 8 to 32 gives about 3% of performance improvement. This denotes that the computation overhead for the chunk partitioning of ReHypar is negligible. However, if the threshold value is too small, then the partitioning to the lower level can be noticeable due to the computation overhead in I/O operations.

**Figure 4:**
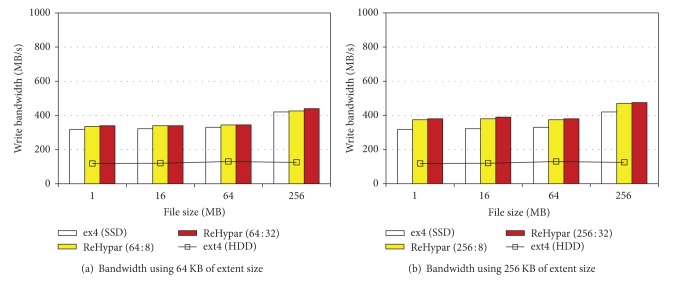
ReHypar write bandwidth as compared to ext4.

Figures 4(a) and 4(b) show I/O bandwidth of ReHypar combined with ext4, while comparing it to ext4 installed on SSD. Although ReHypar generates the better I/O bandwidth as compared to ext4 on SSD, the improvement ratio is smaller than that in ext2. For example, on top of 256 MB of file size, applying 256 KB of extent size to ext2 gives 14% of speedup as compared to ext2 on SSD, whereas applying the same extent size to ext4 results in 11% of speedup as compared to ext4 on SSD.

Also, similar to Figures 3(a) and 3(b), ReHypar with ext4 does not show performance difference between two threshold values; therefore reusing the remaining free space by marking the appropriate threshold value can effectively be executed in the chunk partitioning of ReHypar.

### 4.2. Postmark Experiments

**Figure 5:**
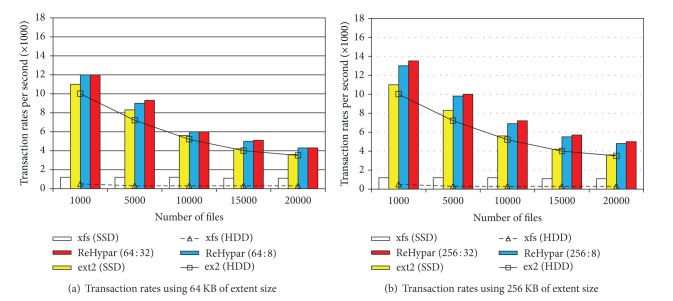
ReHypar transaction rates as compared to ext2 and xfs.

We used Postmark where 100,000 transactions were executed. The number of files increases from 1,000 to 20,000 whose file sizes are varied between 500 B and 10 KB. Figures 5(a) and 5(b) show the transaction rates of xfs and ext2, while comparing to ReHypar integrated with ext2. We set the ratio of read to append operations to (5,5) where read and append operations equally occur.

In the figures, on top of ext2, the performance difference between SSD and HDD is much smaller than that in IOzone write operations. This is because the performance superiority of SSD is offset by generating a large number of small files. In this case, converting into the large I/O granularity on VFS layer can contribute to increase of I/O throughput. For example, with 1,000 files in ReHypar, using 256 KB of extent size generates about 12% higher bandwidth than using 64 KB of extent size. However, the more the files are created, the less the transaction rates are produced because the larger number of inodes is allocated in the same directory, resulting in the memory pressure.

In ReHypar, the effect of the threshold value for the chunk partitioning becomes large with the large-size extent. For example, with 1000 files using 256 KB of extent size, marking *v* as 32 generates about 4% of performance speedup as compared to marking *v* as 8, due to the reduced partitioning overhead.

**Figure 6:**
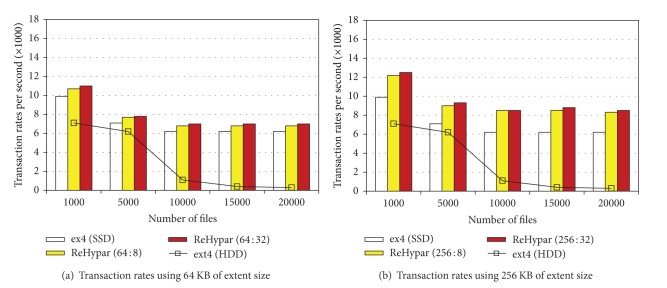
ReHypar transaction rates as compared to ext4.

Figures 6(a) and 6(b) show I/O bandwidth of ReHypar combined with ext4, while comparing to ext4 installed on SSD and HDD. As with ReHypar in Figures 5(a) and 5(b), using the large I/O granularity generates 14% speedup with 1,000 files. Also, the performance difference due to the threshold value is not noticeable, implying that the overhead of the chunk partitioning becomes small in ext4.

## 5. Conclusion

Although SSD is recognized as the next generation storage medium due to its promising characteristics, its high ratio of price per capacity is the main obstacle in replacing hard disk devices. An alternative is to build the hybrid structure where a small portion of SSD address space is combined with the much larger HDD address space. In such a structure, utilizing SSD storage capacity to the maximum extent possible is very important to obtain high I/O performance. Our first objective in developing ReHypar (recursive hybrid chunk partitioning) is to improve the space utilization of SSD partition in the hybrid structure, by reusing the remaining space of I/O units (extents) as much as possible. In order to reuse the unused free space, ReHypar recursively divides the free space using the chunk partitioning method to the lower level and maps the new files to be allocated. Also, ReHypar allows defining several, logical data sections whose extent enables being configured with the different size, to map files to data sections according to file size, usage and access pattern.

We evaluated ReHypar integrated with ext2 and ext4 by using IOzone and Postmark. In those experiments, converting I/O into the large granularity by adopting the large-size extents generates high I/O bandwidth as compared to ext2 and ext4 installed on SSD. Also, the computation overhead to execute the chunk partitioning in ReHypar is negligible; therefore the remaining free space of I/O units can effectively be reused for further file allocations. As a future work, we will verify the effectiveness of the chunk partitioning in ReHypar by using various applications.

## Figures and Tables

**Algorithm 1:**
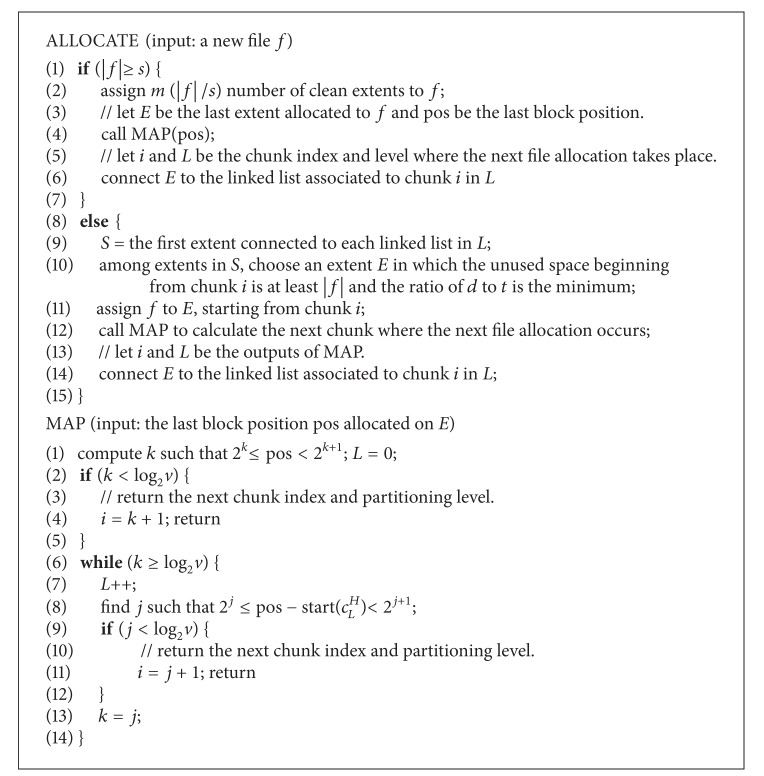
The procedure for the file allocation in ReHypar.

**Table 1:** Extent structure based on threshold (*v*).

Extent size	Threshold (*v*)	Chunk partitioning
64 KB	8	$\begin{array}{c} \phi \left(0,E\right)\overset{\text{level }0}{\to }\left\{c_{0}^{H},c_{0}^{0},c_{0}^{1},\ldots ,c_{0}^{5}\right\}\\ \forall c_{L}^{i}\;\left(i\geq 3,0\leq L\leq 2\right),\phi \left(\text{start}\left(c_{L}^{i}\right),c_{L}^{i}\right)\overset{\text{level }L}{\to }\left\{c_{L+1}^{H},c_{L+1}^{0},\ldots ,c_{L+1}^{i-1}\right\} \end{array}$
	32	$\begin{array}{c} \phi \left(0,E\right)\overset{\text{level }0}{\to }\left\{c_{0}^{H},c_{0}^{0},c_{0}^{1},\ldots ,c_{0}^{5}\right\}\\ \forall c_{L}^{k}\left(k\geq 5,L=0\right),\phi \left(\text{start}\left(c_{L}^{k}\right),c_{L}^{k}\right)\overset{\text{level }L}{\to }\left\{c_{L+1}^{H},c_{L+1}^{0},\ldots ,c_{L+1}^{k-1}\right\} \end{array}$

256 KB	8	$\begin{array}{c} \phi \left(0,E\right)\overset{\text{level }0}{\to }\left\{c_{0}^{H},c_{0}^{0},c_{0}^{1},\ldots ,c_{0}^{7}\right\}\\ \forall c_{L}^{i}\;(i\geq 3,0\leq L\leq 4),\phi \left(\text{start}\left(c_{L}^{i}\right),c_{L}^{i}\right)\overset{\text{level }L}{\to }\left\{c_{L+1}^{H},c_{L+1}^{0},\ldots ,c_{L+1}^{i-1}\right\} \end{array}$
	32	$\begin{array}{c} \phi \left(0,E\right)\overset{\text{level }0}{\to }\left\{c_{0}^{H},c_{0}^{0},c_{0}^{1},\ldots ,c_{0}^{7}\right\}\\ \forall c_{L}^{k}\;\left(k\geq 5,0\leq L\leq 2\right),\phi \left(\text{start}\left(c_{L}^{k}\right),c_{L}^{k}\right)\overset{\text{level }L}{\to }\left\{c_{L+1}^{H},c_{L+1}^{0},\ldots ,c_{L+1}^{k-1}\right\} \end{array}$
